# Luffa operculata effects on the epithelium of frog palate: histological features

**DOI:** 10.1016/S1808-8694(15)31300-8

**Published:** 2015-10-20

**Authors:** Mônica Aidar Menon-Miyake, Paulo Hilário Nascimento Saldiva, Geraldo Lorenzi-Filho, Marcelo Alves Ferreira, Ossamu Butugan, Regiani Carvalho de Oliveira

**Affiliations:** ^1^ Ph.D. in Sciences, University of Sao Paulo (Researcher physician, Discipline of Otorhinolaryngology, HC - FMUSP); ^2^ Physician, Full Professor, Faculty Professor (Laboratory of Experimental Environmental Pollution, Department of Pathology); ^3^ Post-doctorate studies, Laboratory of Experimental Environmental Pollution, Department of Pathology. Discipline of Pneumology - Instituto do Coraçã o (InCor); ^4^ Biologist (Laboratory of Cellular Biology, Department of Pathology); ^5^ Physician, Full Professor (Associate Professor, Discipline of Otorhinolaryngology); ^6^ Biologist (Laboratory of Experimental Environmental Pollution, Department of Pathology)

**Keywords:** luffa operculata, rhinitis, sinusitis, mucociliary clearance, tight junctions, herbal medicine

## Abstract

Luffa operculata is the botanical name of *buchinha-do-norte* or *cabacinha*, which is a medicinal plant widely used for the treatment of rhinitis and rhinosinusitis. In Europe and USA, it is available in homeopathic medicines. In Brazil, Luffa operculata dry fruit infusion is inhaled or instilled into the nose releasing profuse mucous secretion, thus relieving nasal symptoms. Nevertheless, this often may cause irritation, epistaxis or anosmia.

**Study design:**

Experimental.

**Material and Method:**

The effects of Luffa operculata were evaluated in different concentration infusions, in isolated frog palate preparation, testing 46 palates after immersion. Four groups (n = 10) were tested with the infusion prepared with frog Ringer (isotonic): control; 60 mg/L; 600 mg/L; and 1200 mg/L. An additional group was tested using the infusion with water (600 mg/L H2O, n = 6). Epithelial samples were harvested to be studied under light microscopy and electron transmission microscopy.

**Results:**

In treated palates, light microscopy findings were dose-dependent standard toxic changes. Electron transmission microscopy showed enlargement of intercellular spaces and tight junctions disruption, pointing to ion-fluid transport abnormalities. **Conclusions**: Luffa operculata infusion in currently used doses can promote significant structural and ultrastructural changes in the epithelium of this ex vivo model of respiratory mucosa.

## INTRODUCTION

Luffa operculata, known in Brazil as *buchinha-do-norte* or *cabacinha*, is the most widely recalled and used medicinal plant by the population to treat rhinitis and rhinosinusitis^1^. Despite the lack of scientific evidence about its use, most ENT physicians know of its effects and receive many patients that had used the plant (although some do not report it), presenting adverse reactions such as epistaxis, nasal irritation and olfaction affections.

The medicinal use of Luffa operculata and other medicinal herbs is part of a larger context: this practice has grown all over the world for the past decades in an attempt to find alternative medicine practices, which is scientifically named complementary medicine^2^. Medicinal plant use is an ancestral and disseminated form of medicine. In Brazil, it is traditionally employed by poor rural populations, but medicinal herbs have invaded urban centers, and are being used by patients of all social-economic and cultural background. The phytotherapic market negotiates billions of dollars annually in Europe and North America^3^. A significant portion of users of complementary medicine tries to find treatment for respiratory diseases such as allergic rhinitis, rhinosinusitis and asthma^4^.

Distortions resulting from inappropriate use of medicinal plants are related with the mistaken belief that treatment with natural plants never makes any harm. There may be problems resulting from poor origin of the plant, inappropriate storage, inadequate dose and preparation, and even drug interaction that may take to peri-operative complications^5^, ^6^, a fact that should be taken into account by all specialists in surgical areas. In the case of Luffa operculata, it is possible to cause worsening or complication of rhinosinusitis without anyone associating it with the use of the plant.

### Luffa operculata

**Figure 1:**
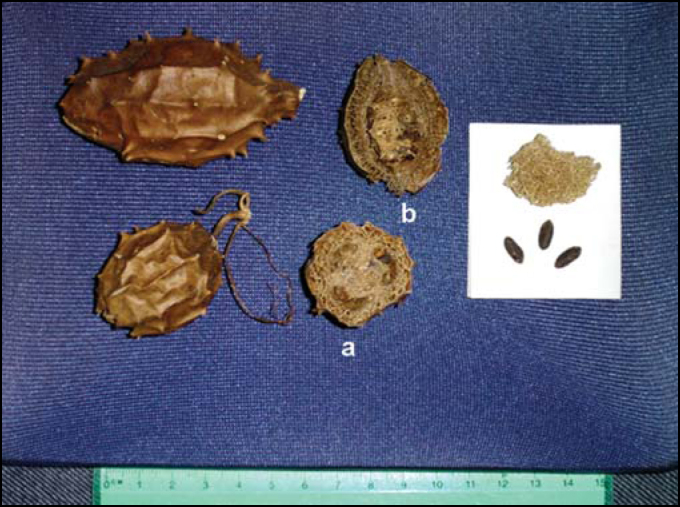
Dry fruit of Luffa operculata, transversal (a) and longitudinal (b) sections. The detail shows the fibrous mesocarpus and the seeds.

Luffa operculata is a dicotyledonous of family Cucurbitaceous. Among the many popular names, it is known in Brazil as buchinha-do-norte or cabacinha, and esponjuelo or esponjilla in Latin America (Figure 1). Its use has been in place for over one century. “Purgative pills made of potato resin and momordica by Surgeon Mattos”, or *pilula do mato* were used for years as the preferred plant agent in rural areas of North and Northeast regions of Brazil. Aqueous extract of Luffa operculata in association with other herbs originate *garrafada*, a mixture known in the countryside and Northeast region of Brazil as being abortive and purgative, demonstrating the powerful irritating action over the mucosa. The market currently offers a formal preparation based on Luffa operculata 1% and sterile solutions, for free commercialization in drugstores.^7^

To treat rhinitis and rhinosinusitis, the population uses dry fruit of Luffa operculata in a well-known recipe that is informally recommended by medicinal herb salespeople. They recommend infusion of ¼ of the fruit in 500ml of water administered by inhalation or instillation of nasal drops, followed by profuse mucupurulent rhinorrhea, sometimes with bloody aspect, followed by expelling of polyps^8^, ^9^. Another way of preparing it is by washing in 9 waters^8^. The toxic dose of Luffa operculata in humans, extrapolated from LD50, corresponds to 170mg/Kg. Thus, approximately 1g of extract of Luffa operculata may be lethal for a 70Kg adult^10^.

Luffa operculata acts on mucosas thanks to the effects of cucurbitacines and glycosides. Saponin collaborates with the action, emulsifying active liposoluble compounds, which facilitates contact and absorption of isocucurbitacine through the mucosas and results in caustic action over them^8^. Few experimental or clinical studies have been published with drugs based on Luffa operculata^7^, ^11^, ^12^, ^13^, and one of them assessed epithelial ultrastructure^14^.

### Form and Function of Respiratory Epithelium

The nasal mucosa is predominantly constituted of pseudo-stratified ciliate cylindrical epithelium and produces mucus, whose transport us taken cephalo-caudally. Mucociliary transportation occurs thanks to rheological properties of mucus (that is, its viscoelasticity), cilia in metachronic waves and coupling between cilia and mucus. These characteristics depend on morphology of epithelium and lamina propria^15^.

The respiratory epithelium separates the lumen of interstitial components and forms a barrier, such as a blanket of cells strongly adhered by tight junctions. The main role of these structures is to inhibit the movement of water and solutes through the epithelium, forming a barrier to the passage of ions and molecules through paracellular pathway, and movement of proteins and lipids between basolateral and apical domains of plasma membrane. Over 40 different proteins constitute the tight junction. This term is widely used in the literature and has received a number of translations to Portuguese, but the preferred use is of the original term in English^16^, ^17^.

### Experimental Model of Isolated Frog Palate

It is a convenient system for the study of nasal mucosa and mucociliary system, because the epithelium is virtually identical to the airways of mammals^18^. The amphibian presents the palate recovered with pseudo-stratified ciliated columnar epithelium, recovered by mucous film^19^. It continues to secrete mucus and maintains ciliary beating for up to 14 days after sacrifice of the animal, if maintained under refrigeration. It may happen as a result of mimicking of the conditions of frogs in their natural habitat during hibernation periods. Rana catesbeiana, known as *bullfrog*, has mucus with viscoelastic properties similar to that of normal human beings^20^.

In view of the lack of data over the action of Luffa operculata on respiratory epithelium, we conducted a dose-response study with infusion of Luffa operculata, starting from the empiric dose normally recommended in an experimental animal model with frog isolated plate, to assess qualitative histological aspects of frog palate, under light microscopy and transmission electron microscopy.

## MATERIAL AND METHOD

The present study was conducted at the Discipline of Otorhinolaryngology and Laboratory of Experimental Environmental Pollution (Department of Pathology) between February 2002 and June 2003. It was approved by the Ethics Committee for Research Projects of Hospital das Clinicas, Medical School, University of Sao Paulo.

We studied 46 isolated palates of frogs, distributed into 5 groups:

Group 1 (or control group): ten palates examined after immersion in Frog Ringer.

Group 2 (or diluted infusion): ten palates examined after immersion in infusion prepared with 1/4 dry fruit in 5 liters of Frog Ringer (60mg/l or 0.3g/5000ml).

Group 3 (or infusion base): ten palates examined after immersion in infusion of Luffa operculata prepared with 1/4 dry fruit in 500ml of Frog Ringer (600mg/l or 0.3g/500ml).

Group 4 (or concentrated infusion): ten palates examined after immersion in Luffa operculata infusion prepared with 1/4 dry fruit in 250ml de Frog Ringer (1200mg/l or 0.3g/250ml).

Group 5 (or water infusion): six palates examined after immersion in infusion of Luffa operculata prepared as the empirical popular formula: 1/4 dry fruit in 500ml of mineral water (600mg/l H20 or 0.3g/500ml H20).

We used frogs of species Rana catesbeiana (bullfrog), maintained in the animal lab of the institution, coming from frog raising facilities. Each frog was sacrificed by decapitation after having being one hour in a recipient with ice, which leads to low metabolic rate, reducing reflexes and mobility, in addition to physical anesthesia in the animal to minimize animal suffering. After decapitation, mandible was disarticulated and the upper portion of the head was removed.

The palate was placed on Petri dish over gauze soaked with Frog Ringer, covered by plastic film and preserved under refrigeration (4°C) for 2 days. In such conditions, the ciliary activity is maintained.

The preparation of Luffa operculata infusion was empirically based on the proportion observed in the popular formula, as provided by salespeople that commercialize the plant: ¼dry fruit in 500ml water. The diluted infusion seems to be a homeopathic drug and the concentrate formulation requires the preparation of infusion with double the amount of the fruit. The infusion is the aqueous extract obtained from vegetable matter, placed in boiled water and later closed with a lid, similarly to what we make with coffee or tea. The amount of fruit mass was standardized to minimize the variation of quantity of active principle in the experiment. We included in the preparation of infusions, at different concentrations, part of the peel, fibrous pulp and seeds of Luffa operculata. We weighted 10 dry fruits of Luffa operculata with approximate variation of 1g to 1.5g, and mean of 1.2g. The value was defined as standard weight of a fruit for the experiment. The solution is made with ¼ dry fruit, which corresponds to 0.3g. We defined the concentrations of each of the solutions of infusion based on this weight, as described above.

To assess the action of Luffa operculata isolated in an animal experimental model, the base of the studied infusion was sterile solution for frogs, Frog Ringer, which is a 1:1 dilution of Ringer in sterile solution. A group was studied using water as solvent and the results were compared with the other groups.

Before collecting the material for histological analysis, the study included assessment of mucociliary activity of frog palate epithelium with three different parameters: speed of mucociliary transportation, frequency of ciliary beating, difference in transepithelial potential^21^, ^22^. The palates for each group of study were examined three times: before immersion in infusion of Luffa operculata, after 5 minutes of immersion and after 20 minutes of immersion, times determined as a result of nasal clearance of saccharin in humans. At the end of the tests, we collected a sample of the epithelium for histological analysis, and the total for each palate was 25 minutes in contact with the studied solution, with time intervals for rehydration in nebulization with Frog Ringer.

For histological analysis, we collected samples of the epithelium of all palates, fixed in buffered formaldehyde solution 4% for histological processing in paraffin blocks. Slides were divided and stained with hematoxyilin-eosin (HE) to analyze the characteristics of the epithelium and lamina propria under light microscopy and solution of periodic acid of Schiff and Alcian Blue (PAS/AB). The latter stains in red any neutral mucus (PAS+) and in blue any acid mucus (AB+). We used microscope ZEISS.

We collected samples of two palates from each group, fixed in glutaldehyde 2% and post-fixed with osmium tetroxide 1.3% to study the ultrastructure under transmission electron microscopy. We used electronic microscope JEOL 1010, with magnification of 8,000 to 80,0000 times. This step in the work was conducted at Laboratory of Cellular Biology, Medical School, University of Sao Paulo.

## RESULTS

Upon making the infusion, we noticed that the more foam formation, the higher the solute concentration, when it came to a boil and was taken off the heat. We studied frog palates treated with different concentrations of infusion of Luffa operculata and observed clear microscopic increase in amount of mucus after immersion, with the presence of water bubbles. The increase was related with concentration of infusion, intensified in the palates treated in groups 3, 4 and 5.

**Figure 2:**
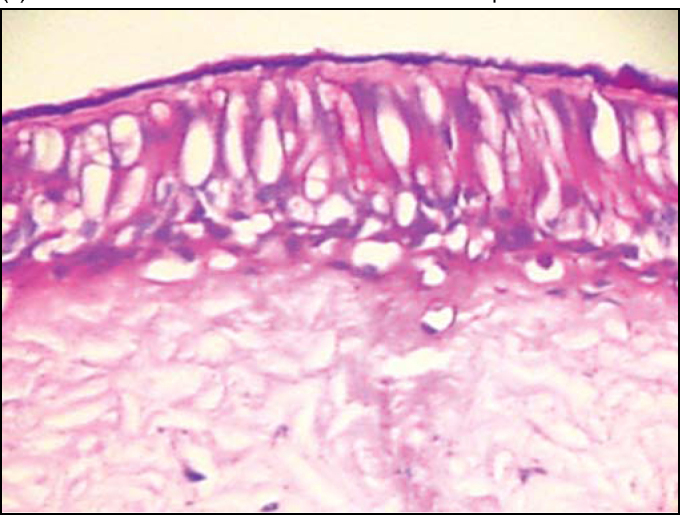
Slide with frog palate epithelium from group 1 showing normal aspect: mucous layer in contact with preserved epithelial surface and lamina propria (light microscopy, PAS-AB staining, 100 times magnified).

**Figure 3:**
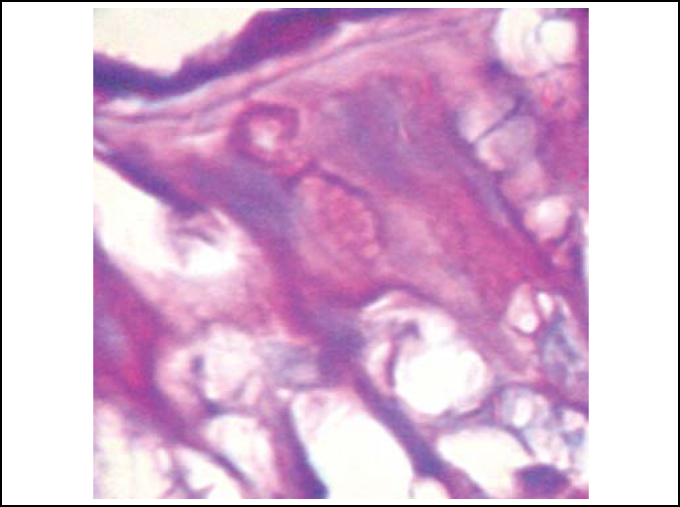
Slide with frog palate epithelium from group 1 showing details of lamina propria glands (light microscopy, PAS-AB staining, 400 times magnified).

**Figure 4:**
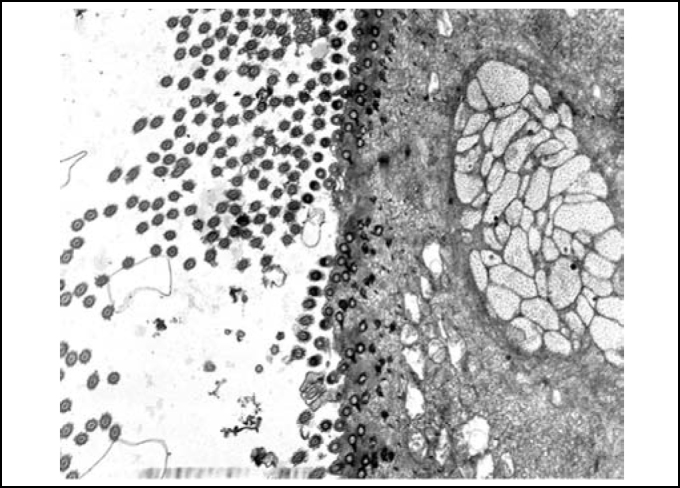
Slide with frog palate epithelium from group 1 showing cilia at transversal section and mucous contained in mucous cell (transmission electron microscopy, 800 times magnified).

**Figure 5:**
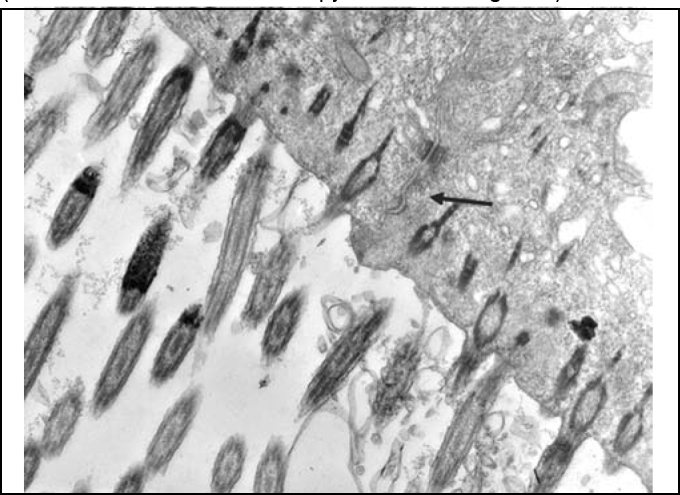
Slide with frog palate epithelium from group 1 showing cilia at longitudinal section and preserved tight junction (arrow) between two contiguous cells (transmission electron microscopy, 20,000 times magnified)

**Figure 6:**
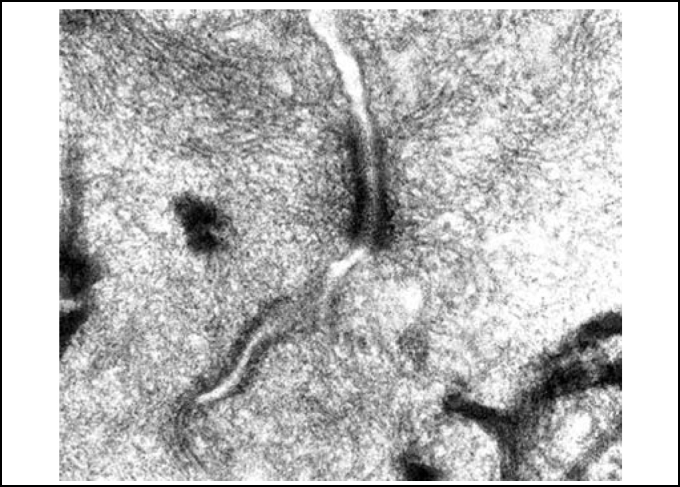
Slide with frog palate epithelium from group 1 showing details of desmosome, surrounded by intermediate filaments of cytokeratin (transmission electron microscopy, 800 times magnified).

In palates in the control group (Group 1), we could evidence integrity of epithelium, with presence of cilia and preserved mucous layer, present submucous and mucous cell without content (Figures 2 and 3). Under electron microscopy, cells were aligned side by side, linked by desmosomes and tight junctions and preserved cilia and basal corpuscle layers (Figures 4, 5 and 6). Palate epithelium in group 2, which corresponds to more diluted infusion, presented histological aspect similar to the control group, with small variations, which could be perfectly seen in Group 3. Abnormalities of the epithelium are gradually more marked and frequent as there is increase in concentration of studied infusion.

Thus, typical areas of preserved epithelium were more rarified the more we selected the material for transmission electron microscopy. Group 4, with concentrated infusion of Luffa operculata in Frog Ringer, had results closed to Group 5, with palates immersed in infusion made with water.

**Figure 7:**
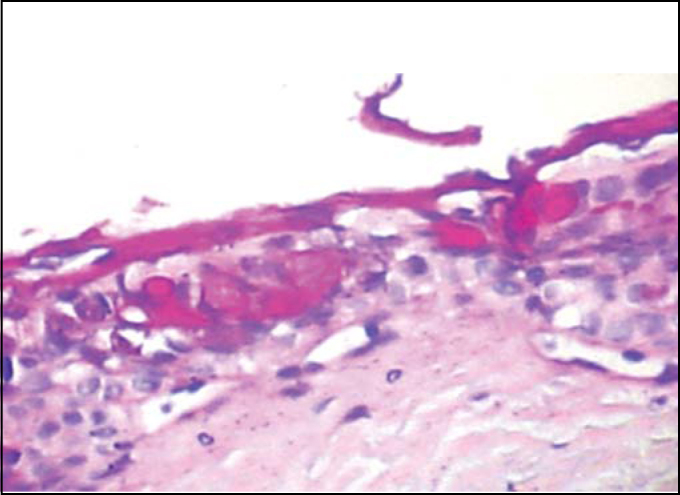
Slide with frog palate epithelium from group 5 showing disorganization of epithelium and increase in volume of extruded mucus (light microscopy, PAS-AB staining, 100 times magnified)

**Figure 8:**
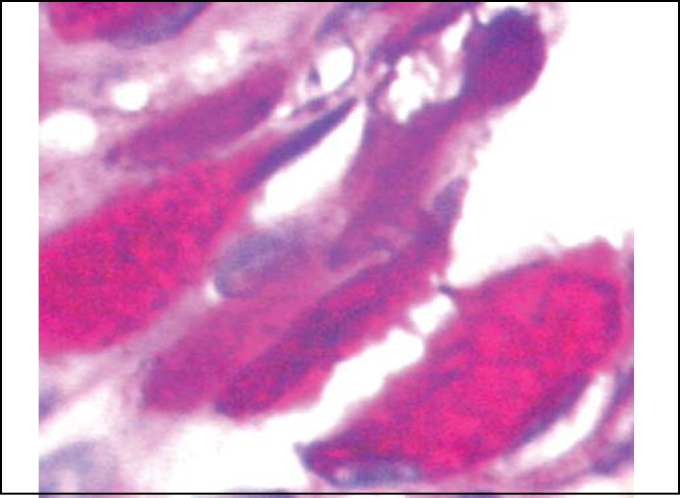
Slide with frog palate epithelium from group 4 showing epithelial destruction, cell vacuolization and increase in intercellular space (light microscopy, PAS-AB staining, 400 times magnified).

**Figure 9:**
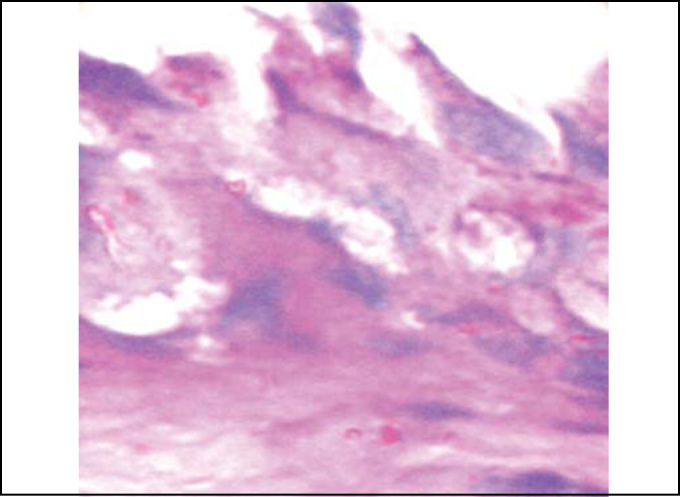
Slide with frog palate epithelium from group 4 showing epithelial disorganization, cell edema and complete extrusion of mucus (light microscopy, PAS-AB staining, 400 times magnified).

In the palates treated with concentrated infusions, we frequently observed, under light microscopy, irregular epithelium with presence of large amounts of mucus in glands and over the epithelium. Sometimes dramatically disorganized, the epithelium presented intercellular edema and grooves, resultant from epithelial rupture (Figures 7, 8 and 9), corresponding to toxicity standard of the epithelium.

**Figure 10:**
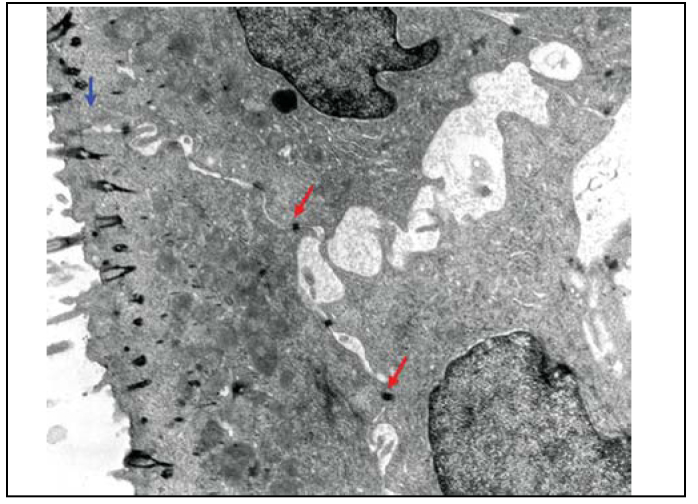
Slide with frog palate epithelium from group 5 showing increase in intercellular space and presence of intercellular junctions: desmosomes (red arrow) and tight junction (blue arrow). The increase in transit of fluid to extracellular matter is morphological evidence of increase in permeability of epithelial barrier (transmission electron microscopy, 10,000 times magnified).

**Figure 11:**
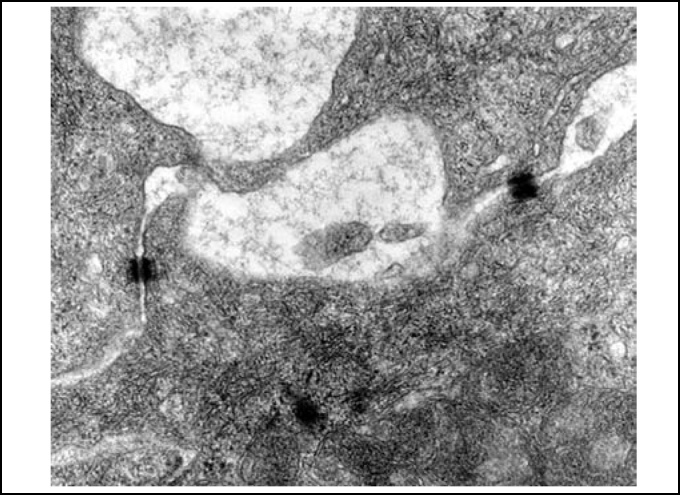
Slide with frog palate epithelium from group 5 showing detail of the slide above, showing two desmosomes and increase in intercellular space with fluid material containing proteins (flocculation) (transmission electron microscopy, 40,000 times magnified).

Upon electron microscopy, we observed loss of cilia and widen intercellular spaces because of intercellular edema, pointing to affections of ionic and fluid transportation (Figures 10 and 11).

## DISCUSSION

Inhalation or nasal instillation of infusion of Luffa operculata (*buchinha-do-norte*) is a very old practice for the treatment of rhinitis and rhinosinusitis, diseases of growing prevalence in the world population^1^, ^8^, ^9^, ^23^. Despite the popular use of Luffa operculata and informal reports of adverse events - epistaxis, nasal irritation, olfaction affections and necrosis of nasal pyramid after prolonged period of use, there are very few studies about the plant. The description of profuse discharge of nasal mucus with the use of Luffa operculata^8^ is the only indication that there is direct effect over the structure and the function of nasal mucociliary system, an aspect that deserved scientific elucidation.

The first step of our study systematically assessed through ex vivo experimental model, the effects of Luffa operculata infusion over three parameters that comprised mucociliary transport: rate of mucociliary transport (indicating integrity of mucus, cilia and their linking), frequency of ciliary beating (system engine) and difference in transepithelial potential (which reflects ionic and water balance of the epithelium).

We observed consistent affections in the three parameters listed above^21^, ^22^. Qualitative morphological study was conducted in the sequence and it confirmed the previous findings, in addition to contributing to the understanding of the action mechanisms of infusion with Luffa operculata on the epithelium and the phenomenon of profuse rhinorrhea caused by Luffa operculata, already observed for decades. It is possible that, in vivo, it may result from mucosa congestion, followed by increased capillary permeability and increase in mucus flow^24^. In our experiment, we detected discharge of intraepithelial mucus contributing to the amount of secretion produced.

The experimental model of frog isolated palate, not very disseminated among Otorhinolaryngologists, proved to be excellent and practical for the assessment of the structure of respiratory epithelium and mucociliary function, with its histological aspects virtually identical to human respiratory mucosa. It has already been used as base for the test of drugs and human mucus in adverse conditions. The limitation of the ex vivo model used is that it did not allow assessment of the influence of the autonomic nervous system or inflammatory reaction observed in vivo.

Owing to the design of the experiment, each palate was in contact with the examined solution for 25 minutes, with time intervals for nebulization with Frog Ringer for rehydration. The tested model was for normal mucosa, but the condition of prolonged exposure to infusion mimics a situation of practice, in which pathological nasal mucosa presents mucociliary clearance affection. We supposed that adverse reactions observed in the clinical practice may be resultant from maximization of Luffa operculata of previous affections of many different pathological conditions, with fragile and modified epithelium by the action of inflammatory process of microorganisms, and drugs such as vasoconstrictors, among others.

Epithelium abnormalities, when observed in a qualitative form under light microscopy and transmission electron microscopy gradually accentuate themselves depending on the concentration of Luffa operculata infusion. Group 5 (water infusion) presented closer results to group 4 (infusion concentrated in Frog Ringer), suggesting that water solvent increases toxicity of the plant to the tissue.

Roncada^14^, using Luffa operculata 0.5% and 1% did not observe significant epithelial affections under electron microscopy after instilling medication for 14 days in the nasal cavity of rabbits. They noticed loss of cilia, also observed in the control group. We emphasized that in the study, the authors used a commercial preparation with Luffa operculata, and not an empirical solution for popular use. In our study, we found epithelial abnormalities in palates treated with Luffa operculata infusion, and there was loss of cilia and intercellular edema. Based on histological findings, we can infer that there is destabilization and rupture of tight junctions and consequently increase in epithelial permeability, which interferes directly on regulation of mucus hypophase. It suggests that concentrated infusion of Luffa operculata affects the integrity of epithelial intercellular junctions and ionic and water balance^25^.

The foam observed in the preparation of infusions may be attributed to presence of saponin in Luffa operculata. Similarly to detergents, there is no emulsification per se, but rather mucosa surface tension affection causing polarization, which results in caustic effect on tissues. Thus, saponins should be the real irritating component. In therapeutic doses, saponins are the active principle of some mucolytic drugs.

It is interesting to point out that there are few studies with Luffa operculata^7^, ^11^, ^12^, ^13^, and especially with homeopathic drugs. The main dilution of components in these drugs do not enable us to state that the result obtained may be attributed to the presence of Luffa operculata.

This study was designed within the context of the increasing interest of the population for complementary medicine practices. Even though medical science evolves a lot, the population attributes medicinal powers to plants that have never been object of a scientific study, mistakenly considered to be innocuous. It is high time that universities and research centers stopped ignoring complementary medicine and medicinal plants. In a country such as Brazil, with the largest biodiversity in the planet, poor in financial resource and extremely rich in cultural diversity, probably the best contribution scientists may give is to retrieve old traditions and provide their scientific validation.

## CONCLUSIONS

We concluded that infusion of dry fruit of Luffa operculata in tested concentrations modifies the morphology of mucociliary epithelium in isolated palate of frogs. Modifications were dose-dependent and occurred in popularly used concentrations. We observed disorganization of epithelium and ultrastructural modifications such as intercellular edema by rupture of tight junctions.
